# Prognostic Markers of Adverse Outcomes in Acute Heart Failure: Use of Machine Learning and Network Analysis with Real Clinical Data

**DOI:** 10.3390/jcm14061934

**Published:** 2025-03-13

**Authors:** Dmitri Shchekochikhin, Kristina Charaya, Alexandra Shilova, Alexey Nesterov, Ekaterina Pershina, Andrei Sherashov, Sergei Panov, Shevket Ibraimov, Alexandra Bogdanova, Alexander Suvorov, Olga Trushina, Zarema Bguasheva, Nina Rozina, Alesya Klimenko, Varvara Mareyeva, Natalia Voinova, Alexandra Dukhnovskaya, Svetlana Konchina, Eva Zakaryan, Philipp Kopylov, Abram Syrkin, Denis Andreev

**Affiliations:** 1 Functional and Ultrasound Diagnostics, Department of Cardiology, Sechenov University, 8 Trubetskaya Str., Moscow 119991, Russia; agishm@list.ru (D.S.); pershina86@mail.ru (E.P.); panov0908@gmail.com (S.P.); sheva7864@gmail.com (S.I.); doc.aabogdanova@gmail.com (A.B.); konchinas@gmail.com (S.K.); evaja44@gmail.com (E.Z.); kopylov_f_yu@staff.sechenov.ru (P.K.); syrkin_a_l@staff.sechenov.ru (A.S.); dennan@mail.ru (D.A.); 2 City Clinical Hospital No.1, 8 Leninsky Ave., Moscow 119049, Russia; a.s.shilova@gmail.com (A.S.); drcor@mail.ru (A.N.); lelbseek@gmail.com (O.T.); zaremabguasheva@gmail.com (Z.B.); rozina.nina@gmail.com (N.R.); aaklimenko@yandex.ru (A.K.); voinovadr@yandex.ru (N.V.); ashmotkina@gmail.com (A.D.); 3 World-Class Research Center “Digital Biodesign and Personalized Healthcare”, I. M. Sechenov First Moscow State Medical University, 8 Trubetskaya Str., Moscow 119991, Russia; suvorov_a_yu_1@staff.sechenov.ru; 4 Ministry of Health of Russia, N.I. Pirogov Russian National Research Medical University, 1 Ostrovitianova St., Moscow 117513, Russia; m.varvara07@yandex.ru

**Keywords:** acute heart failure, emergency department, triage, graph neural network, gradient boosting, artificial intelligence

## Abstract

**Background**: Acute heart failure (AHF) is one of the leading causes of admissions to the emergency department (ED). There is a need to develop an easy-to-use score that can be used in the ED to risk-stratify patients with AHF and in hospitalization decisions regarding cardiac wards or intensive care units (ICUs). **Methods**: A retrospective observational study was conducted at a city hospital. The data from the presentation of AHF patients at the ED were collected. The combined primary endpoint included death from any cause during hospitalization or transfer to an intensive care unit (ICU) for using inotropes/vasopressors. Feature selection was performed using artificial intelligence. **Results**: From August 2020 to August 2021, 908 patients were enrolled (mean age: 71.6 ± 13 years; 500 (55.1%) men). We found significant predictors of in-hospital mortality and ICU transfers for inotrope/vasopressor use and built two models to assess the need for ICU admission of patients from the ED. The first model included SpO_2_ < 90%, QTc duration, prior diabetes mellitus and HF diagnosis, serum chloride concentration, respiratory rate and atrial fibrillation on admission, blood urea nitrogen (BUN) levels, and any implanted devices. The second model included left ventricular end-diastolic size, systolic blood pressure, pulse blood pressure, BUN levels, right atrium size, serum chloride, sodium and uric acid concentrations, prior loop diuretic use, and pulmonary artery systolic blood pressure. **Conclusions**: We developed two models that demonstrated a high negative predictive value, which allowed us to distinguish patients with low risk and determine patients who can be hospitalized and sent from the ED to the floor. These easy-to-use models can be used at the ED.

## 1. Introduction

Acute heart failure (AHF) is one of the leading causes of hospitalizations in developed countries and is characterized by high in-hospital mortality and frequent readmissions, which causes significant healthcare resource utilization [1]. Hospitalization for AHF begins with the emergency department (ED), where, depending on the severity of the initial clinical presentation, patients with AHF can be hospitalized in cardiac wards or intensive care units (ICUs) [2].

A large proportion of AHF patients may immediately require admission to the ICU from ED due to advanced organ dysfunction or other severe complications [3]. It has been shown that patients with AHF admitted to ICUs have a significantly worse prognosis than those admitted to hospital wards [4]. The latter circumstance poses a difficult task for clinicians in the ED, which consists of correctly identifying patients who really need admission to the ICU.

The heterogeneity of AHF’s clinical manifestations makes the stratification of this condition difficult; this is encountered by even experienced clinicians [5]. Several risk-scoring systems allow us to assess the in-hospital prognosis for AHF upon admission to the ED [6,7,8,9]. These scales have several disadvantages; for example, scales [6,7,8] were developed in 2005–2012, before significant changes in approaches to the treatment of chronic heart failure (CHF). The scale in [9], developed using the Japanese patient registry, may not be suitable for other countries due to differences in patient characteristics and treatment patterns that exist between Japan and other countries. The early identification of low-risk patients at the ED helps reduce unnecessary ICU admissions, and the identification of high-risk patients helps provide them with more intensive monitoring and therapy [2]. Thus, there is now a need to develop an easy-to-use score that can be used in the ED to risk-stratify patients with AHF and guide care.

The interactions between predictors and their relationships with outcomes in medicine are often complex and nonlinear, for which modern machine learning algorithms can provide a solution [10]. It is desirable to predict an unfavorable outcome and immediately hospitalize patients with AHF in the ICU using a well-proven algorithm, such as eXtreme Gradient Boosting (XGBoost), along with an unconventional population-modeling algorithm, such as a graph neural network (GNN). Thus, our study aimed to create an easy-to-use model for EDs for the prognosis of AHF based on modern machine learning algorithms.

## 2. Materials and Methods

### 2.1. Study Cohort and Sample

This single-hospital retrospective observational study was conducted at the city hospital in Moscow, Russia.

This study was approved by the First Moscow City Hospital Ethics Committee (approval ID: 7 of 21 September 2021). Informed consent was waived by the ethics committee due to the retrospective nature of this study.

We reviewed the medical records of all consecutive patients with AHF admitted to our hospital between August 2020 and August 2021 and collected hospitalization data from initial presentation at the ED until discharge or in-hospital death. The inclusion criteria were as follows: AHF, which was diagnosed per the European Society of Cardiology (ESC) guidelines [2] (defined as the new onset or worsening of symptoms and signs of HF due to volume overload requiring urgent hospitalization) and age ≥ 18 years.

The exclusion criteria were as follows: (1) active COVID-19 on admission; (2) acute coronary syndrome (ACS); (3) pulmonary embolism; (4) severe ongoing infection; (5) end-stage liver disease (ESLD); (6) end-stage kidney disease (ESKD); (7) out of hospital cardiac arrest; (8) pregnant or breastfeeding patients.

All patients underwent a complete physical examination and history taking during their stay in the ED and a complete blood test, which was analyzed at the center laboratory of our hospital.

Electrocardiograms (ECGs) were performed at the ED. The ECGs were recorded at a standard paper speed of 25 mm/s and calibration of 10 mm/mV. The QTc duration was measured automatically. An echocardiogram (ECHO) was performed by an expert cardiologist at the ED. The ECHO parameters included biventricular systolic function, left ventricular diastolic function, atrial and ventricular dimension, and systolic pulmonary artery pressure (SPAP).

### 2.2. Outcomes and Variable Definitions

CHF, arterial hypertension, and active cancer were defined as the use of medical treatment for these diseases or based on self-report. Chronic kidney disease (CKD) was diagnosed if the estimated glomerular filtration rate (eGFR) was below 60 mL/min/1.73 m^2^ (Chronic Kidney Disease Epidemiology Collaboration (CKD-EPI) formula). Anemia was defined as hemoglobin < 12 mg/dL in women and <13 mg/dL in men. Atrial fibrillation (AF) was self-reported or according to the ECG data at arrival. Diabetes mellites (DM) was defined as self-reported or newly diagnosed when glycated hemoglobin (HbA1c) was >6.5%. History of stroke/transient ischemic attack (TIA) was based on self-report or head computed tomography/brain magnetic resonance imaging.

Based on previous studies [6,7,8,9,11,12,13], the following potential predictors were selected: age, gender, systolic (SBP) and diastolic blood pressure (DBP), heart rate (HR), AF or flutter, respiratory rate (RR), and ECG data (the presence of left bundle branch block (LBBB), QRS duration, and QTc duration); medical history data (previous diagnosis of CHF, myocardial infarction (MI), DM, stroke, cancer, renal replacement therapy (RRT) on an outpatient basis, percutaneous coronary intervention (PCI), aorto- (CABG) and mammary coronary artery bypass grafting (MCBG), pacemaker (PM), implantable cardioverter-defibrillator (ICD), cardiac resynchronization therapy (CRT), and continuous positive airway pressure (CPAP); blood test data (hemoglobin, hematocrit, platelet count, leukocyte count, lymphocyte count, creatinine, blood urea nitrogen (BUN), uric acid, potassium, sodium, chloride, aspartate aminotransferase (AST), alanine aminotransferase (ALT), lactate dehydrogenase (LDH), total bilirubin, international normalized ratio (INR), fibrinogen, total cholesterol (TC), triacylglycerides (TAGs), C-reactive protein (CRP), procalcitonin, and troponin); and ECHO parameters (left ventricular ejection fraction (LV EF), LV end-diastolic volume (LV EDV), right ventricular (RV), right atrium (RA) and left atrium (LA) dimensions, SPAP, inferior vena cava diameter (IVC), IVC collapse, mitral and tricuspid regurgitation, and peak aortic valve velocity).

The primary endpoint included all-cause in-hospital mortality or, if the patient remained alive, the need for using inotropes/vasopressors.

### 2.3. Statistical Analysis

Statistical processing was performed using the R v4.2 and Python v.3.10 programming languages. The normality of distribution was checked using the Shapiro–Wilk test for quantitative features, and the mean, standard deviation, median, interquartile range, and minimum and maximum values were calculated. The proportion and absolute counts were estimated based on categorical and qualitative criteria. Comparative analysis for normally distributed features was assessed with the *t*-test (2 groups) and using the Mann–Whitney U test for abnormally distributed features (2 groups). Comparative analysis of categorical and qualitative features was performed using the Pearson square criterion. If it was not applicable, the Fisher exact test was used. Survival functions were estimated with the Kaplan–Meier approach, and the log-rank test was used to compare survival distributions. The level of significance was 0.05. Univariate regression analysis using logistic regression was used to assess the association between individual predictors and the outcome. Multivariate modeling was performed using the Python programming language.

The original dataset was randomly split into training (training set: n = 635 (70%)) and internal validation (test set: n = 273 (30%)) cohorts, according to the TRIPOD guidelines [14].

Feature selection was performed using XGBoost and GNN from the training set with the pipeline, including 5-fold stratified cross-validation with shuffling, scaling numeric features, and binarizing categorical features. The median SHAP values from 5 folds were calculated for every feature to select up to the top 10 features with the highest values [15]

Both algorithms (XGBoost and GNN) were used for model building on the training set with the selected features. The first included the pipeline with 10-times-repeated 10-fold cross-validation, the same numeric and categorical data transformations as earlier, the XGBoost classifier, and calibration with isotonic regression during the 10-fold cross-validation process. The second approach included numeric and categorical data preprocessing. Afterward, the data were transformed into a k-neighbors graph (2–5 neighbors were tested) with the calculation of the Euclidean distances between neighbors. The adjacency matrix from the graph was passed into 2 consecutive graph convolutional layers and a 2-linear-layer (with 50% dropout) graph neural network (Pytorch Geometric implementation) for 1000 epochs [16]. The batching and shuffling of the input data was carried out, and the Xavier initialization of the weights was performed. Predictions were calibrated with isotonic regression inside the 10-fold cross-validation. The quality of the resulting models was assessed using the ROC curve, as well as with the calculation of sensitivity, specificity, positive and negative predictive values, and 95% confidence intervals for all the statistics. For both models, the optimal probability cut-off was calculated from predictions on the training data with Youden’s J statistic. Bootstrapping 1000 times and a permutation test were used to compare the calculated AUCs on the test set.

## 3. Results

### 3.1. Cohort Characteristics

**Figure 1:**
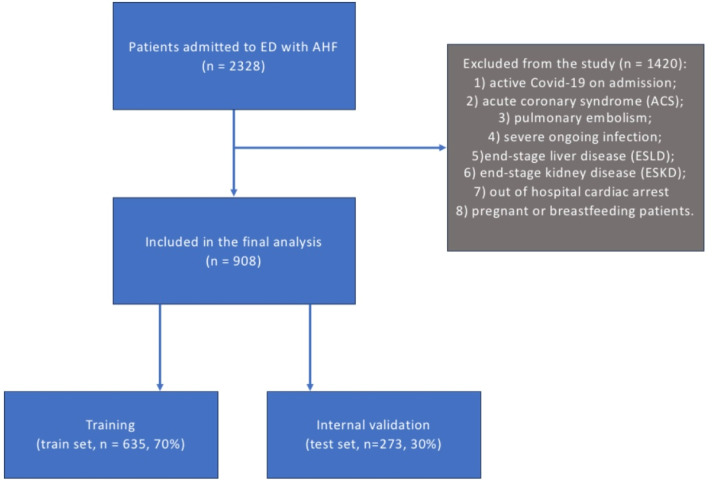
Flowchart of inclusion of AHF patients in this study. AHF—acute heart failure; ED—emergency department.

From August 2020 to August 2021, 908 patients were enrolled in this study. The mean age was 71.6 ± 13 years, including 500 (55.1%) men, and 748 (82.4%) had de novo HF. The characteristics of the patients enrolled in this study are shown in Table 1. A flowchart diagram for this study is shown in Figure 1.

### 3.2. Risk of In-Hospital Outcomes According to Model Predictions

#### 3.2.1. Main Outcome

Among the study cohort, 81 (8.92%) met the primary endpoint. There were 64 (7%) deaths and 17 (1.87%) patients needed to use inotropes/vasopressors.

**Figure 2:**
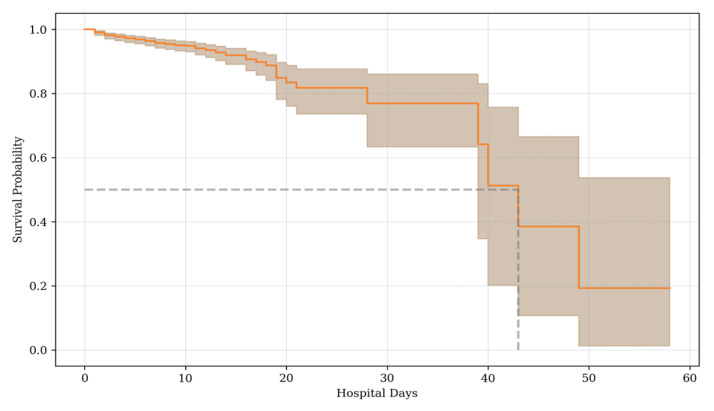
Kaplan–Meier curve for survival in the whole cohort. Dashed line shows median survival time.

The median survival time was 43 days (39; –) (Figure 2).

#### 3.2.2. Feature Selection and Modeling Results

After dividing the data by 70%/30% for training and validation, two cohorts were formed (Table 2).

**Figure 3:**
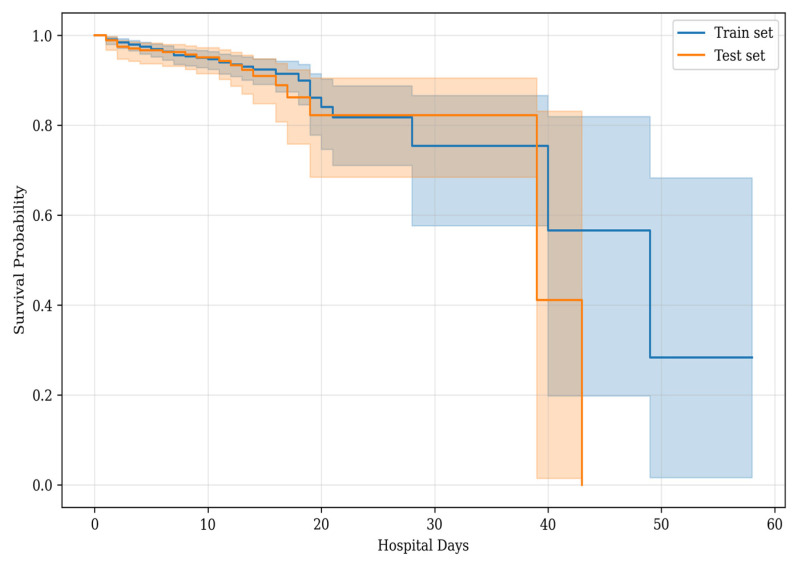
Kaplan–Meier curves for survival in the training and test sets. The non-significant results of the log-rank test show the equality of the survival distributions.

The log-rank test showed no difference in the survival distributions between sets (*p* = 0.27). The Kaplan–Meier curves in both sets were similar, as shown in Figure 3.

**Figure 4:**
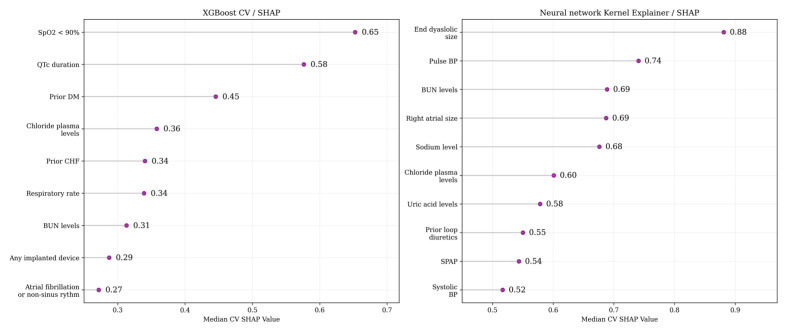
Top features selected, with both algorithms using cross-validation and SHAP value calculation.

Potential predictors were selected by estimating the SHAP values for the training set based on the median SHAP values after five-fold cross-validation. The top predictors with the highest median SHAP values are presented in Table 3 and Figure 4, including both clinical, demographic, and laboratory test results.

The selected predictors were used to subsequently build a classification pipeline using XGBoost and a GNN. After building both pipelines, the models were validated on a test set. The results are presented in Table 4 and Table 5.

**Figure 5:**
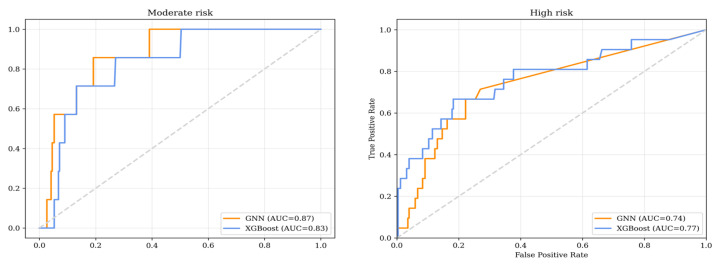
Receiver operator curves for the received prognostic model.

Both models demonstrated a high negative predictive value, allowing us to select factors that were more associated with the exclusion of an unfavorable outcome (Figure 5).

**Figure 6:**
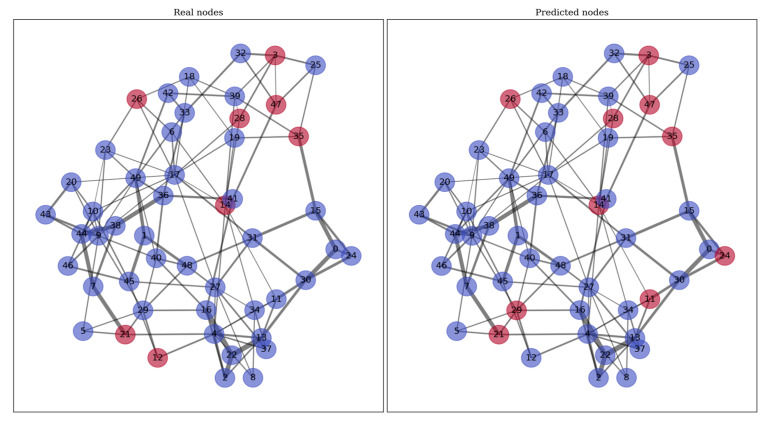
Representation of the patient cohort as a network.

The logic of representing patients as a network can be visualized as follows (Figure 6).

A fragment of the test set is visualized in Figure 6, where each patient is a node of the network and is connected to ‘neighboring’ patients that have similarities based on selected features via SHAP. The part representing real nodes shows patients who died during hospitalization with red. The part with prediction shows patients selected by the neural network with a probability of risk of death above the cut-off calculated during training. The thickness of the lines correlates with the Euclidean distances between neighboring patients. The “Real nodes” graph shows patients who died during hospitalization with red or survived (purple nodes); the “Predicted nodes” graph shows patients with high (red) or low (purple) risk of death.

## 4. Discussion

Physicians in the ED face the difficult task of providing urgent diagnostic and therapeutic procedures and making disposition decisions [17]. However, patients presenting to the ED with AHF are heterogeneous in their physical examinations, burden of comorbidities, and prognoses, which significantly complicates the work of doctors in the ED as decision-makers regarding whether to hospitalize a patient in the ICU or a general hospital ward [18]. The European Society of Cardiology (ESC) for the diagnosis and treatment of AHF has not addressed specific recommendations regarding the management of AHF patients in the ED but emphasizes that AHF treatment is time-sensitive and should begin as early as possible [2]. Thus, there is a need for a scale with rapidly available variables, which would allow a quick assessment of a patient’s prognosis and the coordination of the next steps in care [12].

Using this retrospective registry, we found significant predictors of in-hospital mortality and ICU transfers for inotrope/vasopressor use and built two models to assess the need for ICU admission of patients from the ED. The first model obtained using XGBoost includes SpO_2_ < 90%, QTc duration, prior DM, serum chloride concentration, prior HF diagnosis, RR, BUN levels, any implanted device, and AF on admission to ED. The second model obtained using GNN includes LV EDS, PBP, BUN levels, RAS, serum chloride, sodium and uric acid concentrations, pulse BP, SBP, and SPAP. The data for all the variables were collected at admission to the ED. Both models demonstrated a high negative predictive value, allowing us to distinguish the patients with a low mortality risk. This may be due to the relatively small number of patients with a fatal outcome and because the primary method of feature selection was uniform for both pipelines. Thus, the selected factors are more associated with the exclusion of an unfavorable outcome.

One of the factors associated with an unfavorable in-hospital prognosis in our study was QTc duration. It is known that the QTc interval reflects the duration of ventricular depolarization and repolarization and is an important indicator of normal cardiac function [19]. A prolonged QTc duration is an established prognostic marker in patients with CHF and is associated with worsening symptoms and all-cause mortality [20,21]. Finally, the study in [19] showed that AHF patients with prolonged QTc durations have a higher risk of short-term adverse events. Our results suggest that measurement of the QTc duration in a 12-lead ECG is an easily available way to determine the complement of one factor of unfavorable prognosis in AHF.

Physical examination is a critical component of assessment in ED, and BP measurement is a simple procedure performed in all patients with AHF. Traditionally, patients with AHF are classified as hypertensive (with SBP > 140 mm Hg), normotensive (SBP 100–140 mm Hg), or hypotensive (SBP < 100 mm Hg) [22]. It is known that low admission SBP is an independent predictor of in-hospital death in AHF [22,23,24], which was reflected in our study. BP reflects cardiac reserve and systemic resistance and largely determines the possibilities and tactics of treatment in AHF [22]. Most patients with AHF are classified as hypertensive [23]. The hypertensive form of AHF usually manifests with a more dramatic clinical picture and severe symptoms, but these patients have lower mortality rates compared with patients with low BP and are easier to treat [22]. The hypotensive form of AHF is challenging to manage and is a poor prognostic factor [22], so our results regarding the prognostic value of admission BP reflect previously accumulated data and are expected. Low pulse BP in AHF reflects a decrease in stroke volume and is an independent predictor of mortality, as has been shown in studies [25,26]. Low pulse BP as an unfavorable prognostic sign was reflected in one of the scales we obtained.

Calculation of RR is part of the physical examination in ED, and tachypnea is a nonspecific symptom that may accompany AHF and indicate a poor prognosis [27]. Because dyspnea is one of the most common complaints in AHF, pulse oximetry-derived oxygen saturation (SpO_2_) determination is part of routine patient assessment in the ED [28]. The most common causes of a fall in blood oxygen concentration (hypoxemia) in AHF, defined as SpO_2_ < 90%, are pulmonary edema (PE), cardiogenic shock (CS), and associated conditions, such as pneumonia, chronic obstructive pulmonary disease (COPD), bronchial asthma (BA), and other concomitant lung diseases that can significantly affect the prognosis of patients with AHF [29]. Hypoxemia with SpO_2_ < 90% affects the management of patients with AHF since, in this case, supplemental oxygen is required with a possible transition to non-invasive lung ventilation (NIV) [29].

According to the results of our analysis, it was the BUN level, and not blood creatinine and eGFR, that had a significant impact on the in-hospital prognosis in AHF. High BUN levels on admission are a known adverse factor in patients hospitalized with AHF [30]. BUN is a marker of neurohumoral activation and a poorer response to therapy, a higher adverse events rate, and a higher all-cause mortality rate [30]. Our results show the need for increased vigilance regarding the management of patients with high BUN levels on admission.

In AHF, elevated uric acid levels are associated with higher daily doses of loop diuretics, and hyperuricemia at admission is associated with higher mortality [31]. A meta-analysis [32] demonstrated the adverse prognostic impact of high blood uric acid levels in AHF.

In the AHF setting, a low serum sodium level is a well-known predictor of adverse outcomes [33,34]. Like increased BUN, hyponatremia in AHF reflects the activation of neurohormonal factors, particularly vasopressin and the sympathetic nervous system (SNS) [35]. Low blood chloride levels in patients with AHF on admission are also a known unfavorable prognostic factor [36], as well as a predictor of the development of hyponatremia during hospitalization [37].

DM is a common comorbid condition of CHF and is associated with higher hospitalization rates and mortality [38]. DM was associated with an increased risk of in-hospital mortality in AHF in a study [39]. In our study, DM also influenced in-hospital prognosis and was included as a variable in one of the resulting scales. In addition to common pathophysiological links, DM and CHF are characterized by similar comorbidities, which significantly aggravate the course of AHF [38].

Another significant factor in our study was the presence of AF at admission. According to different studies, the influence of AF on in-hospital prognosis is controversial [40]. From a pathophysiological point of view, AF can be a triggering factor of AHF or a consequence of AHF [41]. The presence of AF upon admission determines additional medical actions, such as electrical cardioversion and the prescription of antiarrhythmic drugs and anticoagulants, which can also affect the prognosis [2,41].

Assessment of cardiac chamber volumes is a mandatory component of the examination of patients with CHF [2]. The presence of LV dilation (measured by LVEDV) is a factor in CV events in patients with CHF [42]. Right atrium (RA) enlargement [43] and pulmonary hypertension derived by ECHO [44] have been associated with a poor prognosis in both CHF and hospitalized patients. All these echocardiographic findings, as well as the presence of implantable devices [2], are markers of more advanced cardiomyopathy and showed prognostic value in AHF in our study.

A history of CHF also adversely affected the prognosis in our study. Patients with acute decompensated heart failure (ADHF) are more comorbid, respond more poorly to diuretic therapy, and have a poorer prognosis compared with patients with de novo AHF [45], which is consistent with our results.

Compared with its analogs, our study included less data, and the demonstrated results are more hypothesis-forming. Given the small size of the training sample, we decided to abandon the development of a grading system or a ranking scale in favor of the raw predicted probabilities using XGBoost. The demonstrated AUC was higher than 0.7, which allowed us to consider that our results were comparable to analogs. The study in [9] is a fairly good analog, but the data from Japanese registries were used, which requires additional validation in other cohorts. An undoubted advantage is external validation with c-statistic = 0.71. However, the routine use of B-type natriuretic peptide is not always available in many regions. Moreover, the factor selection method was stepwise regression, while we can apply more advanced methods with greater capabilities for factor selection. In another study [8], the data were obtained from a very large cohort of 33,533 patients using the prediction rule method based on the construction of CART trees. Such models are often overfitted or sensitive to outliers in new data. The study allowed for the formation of a visual algorithm for use by clinicians, but the publication only presented the model’s ability to identify risk groups without assessing the individual risk of a patient. This is an excellent descriptive model. In a large study [7], the generalized estimating equations method was used to select predictors and build the final model. The c-statistic was quite high for the training and validation data, at 0.75. Another study [6] demonstrated a high c-index of 0.71–0.73 for different patient cohorts.

In our study, the selection of significant factors was initially carried out using a boosting algorithm with multiple cross-validation, which made the results more robust and restricted the model from overfitting. Factors that have the greatest association with the outcome also attract attention. It was important for us to build a compact model, which enabled us to determine the risk of an outcome with the achievement of the maximum possible AUC for the test sample.

In this study, the construction of a GNN was not inferior to a well-known machine learning algorithm, which has gained wide popularity. The GNN model architecture related to network analysis resembles reality, which was among the aims of the methodological aspects of this study.

From the perspective of clinical guidelines, the data collected from the patients were incomplete. However, in reality, patients are admitted through the emergency channel, which reduces the decision-making time and diagnostic possibilities. In this study, we selected the available predictors. Thus, the concept of explainable artificial intelligence can be operational in real-world settings while being as good as other data classification methods.

## 5. Limitations

This study should be evaluated in light of its limitations. First, this was a single-center retrospective observational study of AHF patients admitted to a single large tertiary center; all score components were collected and calculated retrospectively, and there was a risk of selection bias. Second, the study population was relatively small. Third, a large proportion of the study cohort lacked Nt-proBNP levels, although the latter brings our results closer to real-world practice. Only data from hospitalized patients were used to create both models because the local practice of our hospital at the time of this study was that all patients with AHF were hospitalized. All patients with AHF criteria were hospitalized due to the local practice in 2020–2021 in our hospital. Thus, several patients could be potentially discharged from EDs in other institutions. Our database does not provide follow-up data, so the long-term prognosis of patients in our study is unknown. The decision to transfer a patient to an ICU may depend on many other factors that were not considered in our study, including institutional policies. Finally, it remains uncertain whether the results of this study can be applied to other regions or countries, and further studies with larger numbers of patients are required to confirm our results.

## 6. Conclusions

Based on retrospective data, and using artificial intelligence, we developed two models that can help determine patients with AHF as to who can be hospitalized and sent from the ED to the floor. These easy-to-use models can be used by clinicians from the moment a patient is admitted to the ED.

## Figures and Tables

**Table 1:** Cohort characteristics.

Characteristic	Study Cohort (N = 908)	Met Primary Endpoint (N = 81)	Did not Meet Primary Endpoint (N = 827)	*p*-Value
Medical history				
Age, years	71.6 ± 13	74.9 ± 13.3	71.2 ± 13.0	0.003
Men, N (%)	500 (55.1)	35 (43.2)	465 (56.2)	0.025
De novo HF, N (%)	748 (82.4)	63 (77.8)	685 (82.8)	0.255
Diabetes mellitus, N (%)	346 (38.1)	32 (39.5)	314 (38)	0.786
Hypertension, N (%)	836 (92)	75 (92.6)	761 (92)	0.855
Previous MI, N (%)	563 (62)	51 (63)	512 (61.9)	0.852
PCI, N (%)	340 (37.4)	15 (18.5)	325 (39.3)	<0.001
CABG/MCBG, N (%)	78 (8.6)	4 (4.9)	74 (8.9)	0.219
Valvular heart disease, N (%)	66 (7.3)	5 (6.2)	61 (7.4)	0.691
Prior stroke, N (%)	170 (18.7)	29 (35.8)	141 (17)	<0.001
Prior PM implantation	98 (10.8)	15 (18.5)	83 (10)	0.019
Prior ICD/CRT implantation, N (%)	40 (4.4)	3 (3.7)	37 (4.5)	>0.999
CKD, N (%)	494 (54.4)	63 (77.7)	431 (52.1)	<0.001
Prior dialysis, N (%)	6 (0.7)	5 (0.6)	1 (1.2)	0.430
Cancer, N (%)	46 (5.1)	4 (4.9)	42 (5.1)	>0.999
Prior CPAP, N (%)	16 (1.76)	7 (0.8)	9 (11.1)	<0.001
Vital signs at presentation				
HR, bpm	80 [70; 94]	88.0 [72.0; 102.0]	78.0 [70.0; 92.0]	0.013
SBP, mm Hg	129.7 ± 24.4	113.6 ± 27.0	131.3 ± 23.5	<0.001
DBP, mm Hg	77.2 ± 14.4	68.1 ± 18.0	78.0 ± 13.7	<0.001
Brachial pulse BP, mm Hg	52.5 ± 17.0	45.5 ± 14.9	53.2 ± 17.1	<0.001
RR, brpm	17 [16; 18]	19.4 ± 4.3	17.5 ± 3.1	<0.001
SpO_2_ < 90%, N (%)	106 (11.7)	69 (8.3)	37 (45.7)	<0.001
Admission laboratory values				
Nt-proBNP, pg/mL	4632 [2137; 9172]	5133.5 [2122; 9017]	4115 [2158; 9448]	0.2
Troponin I, ng/mL	0.0 [0.0; 0.0]	0.0 [0.0; 0.1]	0.0 [0.0; 0.0]	<0.001
Creatinine, mcmol/L	123.8 ± 76.7	180.8 ± 125.6	118.3 ± 67.7	<0.001
eGFR, mL/min per 1.73 m^2^	58.1 ± 23.0	42.6 ± 25.6	59.6 ± 22.1	<0.001
Blood urea nitrogen, mg/dL	7.8 [5.9; 11.0]	12.3 [7.7; 21.4]	7.5 [5.8; 10.3]	<0.001
Uric acid, mcmol/L	462.6 ± 178.4	589.2 ± 212.5	450.2 ± 169.8	<0.001
Blood sodium, mmol/l	139.3 ± 4.9	137.1 ± 8.3	139.5 ± 4.4	<0.001
Hyponatremia, N (%)	114 (12.6)	28 (34.6)	86 (10.4)	<0.001
Blood chloride, mmol/l	104.5 ± 6.3	104.6 ± 9.8	104.5 ± 5.9	0.402
Hypochloremia, N (%)	61 (6.7)	8 (9.9)	53 (6.4)	0.234
Blood potassium, mmol/l	4.2 ± 1.1	4.5 ± 2.3	4.2 ± 0.9	<0.001
Hypokalemia, N (%)	132 (14.5)	18 (22.2)	114 (13.8)	0.040
Hyperkalemia, N (%)	26 (2.9)	7 (8.6)	19 (2.3)	0.006
Glucose, mmol/l	6.55 [5.61; 8.23]	7.05 [6.61; 8.44]	5.48 [4.90; 6.51]	0.07
Hemoglobin, g/l	129.4 ± 23.8	126.1 ± 26.4	129.7 ± 23.5	0.134
Hematocrit, %	39.8 ± 9.1	38.6 ± 7.4	39.9 ± 9.3	0.223
Anemia, N (%)	333 (36.7)	296 (35.8%)	37 (45.7%)	0.078
Platelet count, ×10^9^/L	214.0 ± 82.8	195.7 ± 90.6	215.8 ± 81.8	0.004
Leukocyte count, ×10^9^/L	8.1 ± 4.2	10.7 ± 6.6	7.9 ± 3.8	<0.001
Lymphocyte count, ×10^9^/L	21.3 ± 10.2	14.2 ± 9.8	22.0 ± 10	<0.001
Bilirubin, mcmol/L	17.3 ± 13	3.5 ± 1.2	4.2 ± 1.3	0.024
TC, mmol/L	4.1 ± 1.3	3.5 ± 1.2	4.2 ± 1.3	<0.001
TAG, mmol/L	1.4 ± 0.7	1.4 ± 0.5	1.4 ± 0.7	0.420
ALT, U/L	29 [20; 43.6]	31 [25; 69]	29 [20; 43]	0.004
AST, U/L	27 [21.5; 38]	26.4 [21; 36.9]	35 [24; 69]	<0.001
CRP, mg/L	9.6 [2.7; 27.1]	27.9 [9.6; 63.9]	8.7 [2.6; 23.5]	<0.001
Procalcitonin, ng/mL	0.2 [0.1; 0.4]	0.1 [0.1; 0.3]	0.3 [0.1; 0.7]	<0.001
LDH, U/L	301.5 [214.5; 408.5]	362 [241; 611]	293.4 [212; 405]	<0.001
Lactate, mmol/L	2.1 [1.4; 2.9]	2.5 [1.9; 3.6]	2.1 [1.4; 2.8]	<0.001
INR	1.3 [1.1; 1.6]	1.5 [1.2; 1.8]	1.2 [1.1; 1.5]	<0.001
Fibrinogen, g/L	4.2 ± 2.1	4.2 ± 2.2	4.0 ± 1.0	0.428
ECG at admission				
Atrial fibrillation/flutter, N (%)	325 (35.8)	32 (39.5)	293 (35.4)	0.465
LBBB, N (%)	160 (17.6)	20 (24.7)	140 (16.9)	0.080
QRS duration, ms	100 [100; 100]	100 [100; 100]	100 [100; 100]	0.117
Corrected QT interval, ms	500 [400; 500]	500 [400; 500]	500 [400; 500]	0.966
ECHO at admission				
LVEF, %	40.9 ± 12.2	37.1 ± 13.3	41.2 ± 12.0	0.002
LVEF ≤ 40%, N (%)	457 (50.3)	49 (60.5)	408 (49.3)	0.072
LVEF = 41–49%, N (%)	180 (19.8)	14 (17.3)	166 (20.1)	0.649
LVEF ≥ 50%, N (%)	271 (29.8)	18 (22.2)	253 (30.6)	0.149
LVEDV, mL	139.2 ± 65.4	134.6 ± 63.3	139.7 ± 65.6	0.579
LVEDS, mm	5.4 ± 2.4	5.4 ± 2.5	5.3 ± 1.1	0.788
RVD, mm	3.5 ± 1.6	3.5 ± 1.7	3.5 ± 0.8	0.832
LAV, mL	94.9 ± 41.1	94.6 ± 41.1	98.2 ± 40.3	0.304
RAD, mm	5.0 [4.0; 17.5]	5.2 [4.4; 19.1]	5 [4; 17.5]	0.297
RAV, mL	74.2 ± 39.3	86.5 ± 45.3	73.0 ± 38.5	0.004
IVC diameter, mm	2.1 ± 0.9	2.2 ± 0.5	2.1 ± 0.9	0.007
IVC non-collapsable, N (%)	512 (57.5)	61 (75.3)	461 (55.7)	0.001
Mitral regurgitation moderate–severe, N (%)	435 (47.9)	48 (59.3)	387 (46.8)	0.042
Tricuspid regurgitation moderate–severe, N (%)	338 (37.2)	48 (59.3)	290 (35.1)	<0.001
PA SBP, mm Hg	46.9 ± 17.5	53.5 ± 17.5	46.3 ± 17.3	<0.001
PH derived by ECHO, N (%)	705 (77.6)	635 (76.8)	70 (86.4)	0.047
Peak aortic valve velocity, m/s	1.8 ± 1.1	1.9 ± 1.2	1.8 ± 1.1	0.763

ALT—alanine aminotransferase; AST—aspartate aminotransferase; BP—blood pressure; bpm—beats per minute; brpm—breaths per minute; CABG—coronary artery bypass grafting; CKD—chronic kidney disease; CPAP—continuous positive airway pressure; CRP—C-reactive protein; CRT—cardiac resynchronization therapy; DBP—diastolic blood pressure; eGFR—estimated glomerular filtration rate; HR—heart rate; ICD—implantable cardioverter-defibrillator; INR—international normalized ratio; IVC—inferior vena cava; LAV—left atrium volume; LBBB—left bundle branch block; LDH—lactate dehydrogenase; LVEF—left ventricular ejection fraction; LVEDS— left ventricular end-diastolic size; LVEDV—left ventricular end-diastolic volume; MCBG—mammary coronary artery bypass grafting; MI—myocardial infarction; PA SBP—pulmonary artery systolic blood pressure; PCI—percutaneous coronary intervention; PH—pulmonary hypertension; PM—pacemaker; RAD—right atrium diameter; RAV—right atrium volume; RR—respiratory rate; RVD—right ventricular diameter; SBP—systolic blood pressure; TAG—triacylglycerides; TC—total cholesterol.

**Table 2:** Formed cohorts.

Data	N, Included	Moderate Risk, Needed Pressors	High Risk, Died	Median Survival Time, Days
Training set	635	10 (1.6%)	43 (6%)	43 (40, –)
Test set	273	7 (2.6%)	21 (7%)	39 (39, 39)

**Table 3:** The top features selected with both algorithms using cross-validation and SHAP value calculation.

XGBoost CV/SHAP	Neural Network Kernel Explainer/SHAP
SpO_2_ < 90%	LV EDS
QTc duration	PBP
Prior DM	BUN levels
Serum chloride concentration	RAS
Prior HF diagnosis	Serum chloride concentration
RR	Serum sodium concentration
BUN levels	Serum uric acid concentration
Any implanted device	Prior loop diuretics
AF on admission	SBP
	SPAP

AF—atrial fibrillation; BUN—blood urea nitrogen; DM—diabetes mellites; HF—heart failure; LV EDS—left ventricular end-diastolic size; RAS—right atrium size; RR—respiratory rate; PBP—pulse blood pressure; SBP—systolic blood pressure; SPAP—pulmonary artery systolic blood pressure.

**Table 4:** Moderate risk; permutation test for AUC (*p* = 0.448).

Metric	XGBoost	GNN
AUC	0.83 (0.698; 0.931)	0.867 (0.744; 0.951)
Sensitivity	0.714 (0.35; 0.789)	0.857 (0.5; 1)
Specificity	0.774 (0.722; 0.826)	0.665 (0.606; 0.725)
NPV	0.999 (0.976; 0.999)	0.994 (0.982; 1)
PPV	0.077 (0.017; 0.156)	0.1 (0.021; 0.121)

AUC—area under the curve; NPV—negative predictive value; PPV—positive predictive value.

**Table 5:** High risk; permutation test for AUC (*p* = 0.462).

Metric	XGBoost	GNN
AUC	0.744 (0.636; 0.879)	0.765 (0.634; 0.862)
Sensitivity	0.238 (0.056; 0.435)	0.429 (0.211; 0.647)
Specificity	0.996 (0.988; 0.999)	0.937 (0.905; 0.967)
NPV	0.94 (0.91; 0.966)	0.952 (0.924; 0.977)
PPV	0.833 (0.4; 0.999)	0.36 (0.172; 0.556)

AUC = area under the curve; NPV = negative predictive value; PPV = positive predictive value.

## Data Availability

The authors confirm that the data supporting the findings of this study are available from the corresponding author upon reasonable request.
